# Feasibility of a Theory-Based, Online Tailored Message Program to Motivate Healthier Behaviors in College Women

**DOI:** 10.3390/nu14194012

**Published:** 2022-09-27

**Authors:** Patrice A. Hubert, Holly Fiorenti, Valerie B. Duffy

**Affiliations:** Department of Allied Health Sciences, University of CT, Storrs, CT 06269-1101, USA

**Keywords:** mhealth, physical activity, diet, tailored intervention, behavior change theory, Information-Motivation-Behavioral Skills Model, women, young adults, college students, brief intervention

## Abstract

We aimed to test the feasibility of an online survey and tailored message program in young women. Recruited from college campuses, women (*n* = 189) completed an online survey assessing preference for and behaviors toward diet and physical activity as well as theory-based influencers of these behaviors (knowledge/information, motivation, and confidence). Health messages were tailored to the participant’s survey responses and learning style to address misconceptions and motivate or reinforce healthy physical activity and dietary behaviors. Most women reported the survey as relevant (92%) and useful for reflecting on their health (83%), with survey responses variable in level of nutrition and physical activity knowledge, motivation, and confidence. Each woman received four tailored messages—most reported the messages as relevant (80%) and learning new information (60%). Across all messages, nearly half of the participants (~48%) reported willingness to try or maintain healthier behaviors and confidence in their ability. Body size discrepancy and dietary restraint had small effects message responses of information learned, and the motivation and confidence in trying healthier behaviors. In summary, these data support the feasibility of this online tailored message program. The college women found the tailored message program acceptable and useful to motivate healthier behaviors. The findings provide direction for behaviorally focused interventions to improve dietary and physical activity behaviors.

## 1. Introduction

For young adults, the college years present many challenges to the maintenance or development of healthy behaviors (e.g., regular physical activity, high quality diets) [1,2,3,4]. Of particular concern are college women, who face barriers such as lack of knowledge, misinformation, poor body image, social pressures, and time obligations that may cause strain on their motivation and self-efficacy for appropriate and healthy engagement in physical activity and dietary habits [3,5,6,7,8]. These barriers in tandem with the COVID-19 pandemic have caused further negative effects, with more college women reporting significant changes to their physical activity and dietary intake [9,10,11,12,13]. The 2019–2021 American College Health Association surveys found that only 37% of women identifying students engaged in regular physical activity that would qualify them as active adults, 66% reported drinking ≥1 sugar sweetened beverage(s) a day, and only 18% and 32% met the recommended guidelines of consuming 3 servings of vegetables and fruit a day, respectively [14,15,16]. Furthermore, college women may be more apt to have extreme physical activity behaviors to compensate for poor dietary behaviors and vice versa [17,18,19,20,21]. However, failure to develop appropriate and sustainable behaviors can lead to decreased adherence to physical activity guidelines and an overall poor diet quality [7,8,22]. Thus, successful health interventions that provide appropriate information and motivation in this population are warranted.

There has been demonstrated short-term improvements in behaviors in response to health interventions targeting college-aged individuals, however, refinement in methodology and personalization are necessary to improve program quality and outcomes [23,24]. A systematic review found that college students are not engaged with general health promotion messaging, thereby limiting their usability and impact [25]. Personalized approaches, which can involve the tailoring of health information to one’s phenotype, learning preferences, psychosocial characteristics, activity, and environment, are suggested to improve individual effects of health programming [25,26]. Tailoring health information incorporates methods that personalize communication for the intended receiver, assisting in the reading, remembering, and relevancy of information to the participant [27,28]. Tailored communications, versus generalized and generic communications, have demonstrated greater participant benefit to promote and support health behavior change through increased intention and motivation [28,29,30,31,32]. Tailoring of health information to college women may be key in successful marketing of physical activity and dietary messages to motivate healthier behaviors.

The COVID-19 pandemic has highlighted the importance and normalization of online health care and interventions [33,34]. As online interventions in young adults, incorporating tailoring health information into an internet-based program could improve interest, increase accessibility, reduce participant burden, and offer support/feedback [25,30,35,36]. Computer-generated programs offer an efficient way to tailor health messaging [37] and motivate individuals to improve their physical activity and dietary behaviors by automating delivery of messages via text message, email, or social platforms [30,38,39,40]. Automating these methods allows messages to be tailored in response to participant’s self-reported behaviors versus general goals and recommendations to produce greater changes in physical activity and diet [41]. However, due to poor recall and misreporting of physical activity and dietary intake [42], better measures of self-reported behaviors to tailor physical activity and diet messages are warranted [37].

Assessment of individual food preferences and physical activity though surveying likes/dislikes is a feasible way to measure behavior in young adults/college students as it is cognitively simple, less biased by misreporting [43,44,45,46], and has a low time burden [47]. Messages can be tailored to participant reported preferences to help encourage or motivate behavior change. Acknowledging preference and incorporating tailoring into physical activity and nutrition interventions has helped to encourage physical activity engagement [48,49,50,51], increase preference of healthy foods [44,52,53,54,55], and decrease preference for less healthy foods [56,57]. Acceptability and usability of liking surveys with evidenced based tailored messages has been demonstrated in promoting behavior change in children and adolescents [31,32].

The Informational Motivational Behavioral Skills (IMB) Model has been identified as a supportive framework for tailored messages to participant’s behaviors. The IMB Model suggests that each construct (information, motivation, behavioral skills) has a direct effect on behavior; however, behavioral skills mediate the effect of information and motivation on the resulting health behavior [58,59]. This model is commonly used to understand predictive factors for health behavior and outcomes [59,60]. Previous literature supports its use in predicting many different health behaviors, including physical activity and diet [58,60,61,62,63,64,65,66,67,68,69,70,71]. Thus, the IMB model was used to guide further survey development and creation of tailored messages [72].

Our team has used intervention mapping to develop physical activity and diet messages for college students and young adults based on the IMB model [72]. Included in this approach was examination of literature, assessment of previous survey results, and key informant interviews [72]. The messages were designed with simple language and imagery aligned with IMB model to provide information, motivate, and encourage confidence (i.e., behavioral skills) by either reinforcing or motivating behavior change [72]. As these messages were delivered anonymously and one time, confidence was used to operationalize behavioral skills. Messages were evaluated for participant’s response to information, motivation, and confidence as it pertained to the targeted behavior in the message.

Although preliminary feasibility of the survey and tailored messages suggested promising results [72], additional evaluation of feasibility in women is required, as well as testing of information, motivation, and confidence (i.e., behavioral skills) in the survey, response variability, and message usability. Factors such as body size perception and dietary restraint influence women’s health behaviors [17,18,19,20,21] and may influence their response to the messages and impact their motivation or confidence for behavior change [3,5,6,7,8]. Evidence suggests intersecting relationships between body size perception, dietary restraint, diet quality and physical activity in young women, where body size perception or dietary restraint influence eating behaviors, diet quality and physical activity [21,46,73,74,75,76,77,78,79,80,81].

Thus, this study aimed to explore the feasibility of an online tailored message program for young adult college women that aligns with changes in information, motivation, and confidence (IMB constructs). Feasibility was defined as variability in responses to baseline knowledge, information learned, motivation, and confidence as well as acceptability and usefulness of the messages to promote healthier behaviors. Secondly, this study aimed to test the effect of body size discrepancy and dietary restraint on participant responses to the behavioral survey and message evaluation measures. It was hypothesized that body size perception and dietary restraint may influence women’s responses to the tailored messages. The results from this study address the ability of the survey and participant’s response to the messages to provide direction for future health promotion efforts to improve physical activity and diet quality in young women.

## 2. Materials and Methods

### 2.1. Participants

This was an observational, cross-sectional study with a convenience sample of 189 female-identifying college students from multiple campuses of one New England University. The survey was open to all students regardless of gender identity, however for purposes of this study, analysis was limited to only participants who identified as female. Participants were recruited virtually to complete an online survey and tailored message program from February–April 2021.

A key focus of our marketing plan was recruitment of a diverse student population. We employed a comprehensive marketing strategy and outreach with key stakeholders to recruit students of diverse academic interests, demographics, and campus involvement [72]. Key stakeholders for participant recruitment included academic programs and colleges throughout the University main campus and branches, student health and wellness services, student support services, as well as off-campus and commuter student services. Additionally, the research team created a list and contact information of 250 student-run organizations/clubs, with focus on culturally centered groups. A white paper was created that highlighted the study’s purpose, goals, and pictures with brief bios of members of the research team. Prior to initiation of recruitment, research team members reached out to stakeholders and contact persons for each organization to supply them with the white paper, the option to schedule a virtual informational meeting, and identify interest in recruitment assistance efforts. Recruitment information, including the flyer and materials created for social media postings, was sent to the key stakeholders, and interested student groups. In addition, participants were recruited through consistent postings in the online student newsletter throughout the recruitment months [72].

The study received IRB approval from the University Board (X17-084). The online survey began with an information sheet, followed by a yes/no consent to participate. Participation was voluntary, and students could end the online program at any point. After completing, students had the opportunity to enter their email into a raffle for a $25 gift card.

### 2.2. Procedure

This online tailored message program utilized the IMB framework to adapt an evidence-based program, originally conducted with children their parents/caregivers [31] or children in a middle school setting [32], for college students. The program consisted of a validated survey assessing liking/disliking of usual diet and physical activity behaviors [46,82,83], questions assessing current health knowledge and behaviors [72,84,85,86,87,88], and tailored messages driven by response to the liking survey (food and physical activity), intuitive eating, stress, and sleep. Following the IMB framework, the program assessed knowledge/information of participants through: (1) baseline knowledge related to message Information and responses to each message; (2) reported Information learned; (3) Motivation on how much they would like to try/continue targeted behavior; and (4) Behavioral Skills by assessing confidence/self-efficacy to try/continue the targeted behavior.

The program was designed to be conducted online in a single session via an anonymous Qualtrics platform (Provo, UT, USA). After an online assent to participate, students were asked to report demographic information, liking/disliking of foods and activities, health, and diet related questions (including body size perception, dietary restraint, intuitive eating, food insecurity, weight stigma and perception, stress, and sleep), and the usefulness and acceptance of the survey. Students then received their health messages tailored to their responses and responded to a series of usefulness and acceptance questions for the messages individually and collectively.

### 2.3. Socio-Demographic and Health Characteristics

Students were asked to report their year in college, gender identity, age, ethnicity, race, self-reported weight, and height (used to calculate BMI), and current/ideal body size (Figure Rating Scale [84], self-reported eating disorder (yes/no), school or college, and device used to take the survey. Additional health questions surveyed frequency of physical activity, food group consumption, and level of dietary restraint.

**Figure 1:**
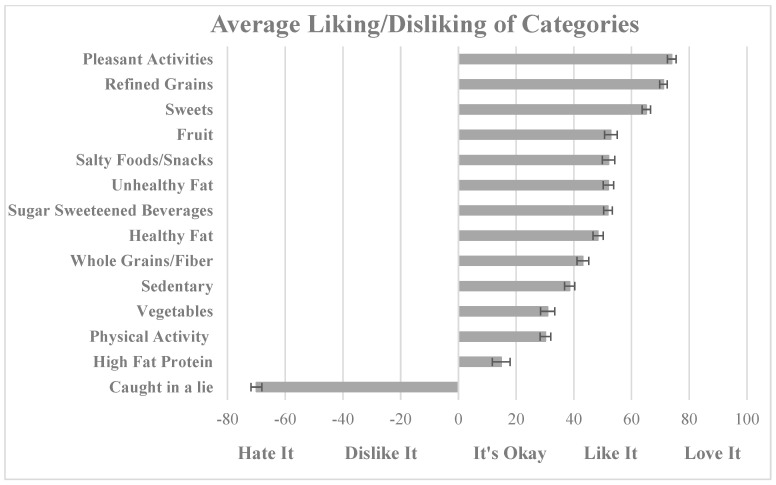
Average reported liking/disliking of foods and activities ranked most to least in young adult college women (*n* = 189).

**Figure 2:**
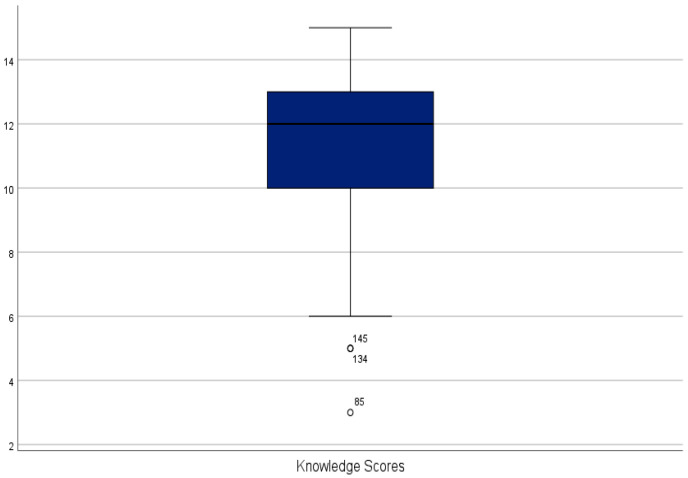
Box Plot of knowledge scores.

Body Size Discrepancy: Participants responded twice to Figure Rating Scale [84,89] to choose which figure represented what they consider their current and then ideal body. The Scale consists of 9 figures (males and females) representing underweight to obese body types [84,89], including figures 1–2 as underweight, 3–4 as normal weight, 5–6 as overweight, and 7–9 as obese. The body size discrepancy variable used in the analysis was ideal body figure subtracted from current figure as a proxy of body dissatisfaction [73,74,90,91]. The variable was treated continuously to test relationships with responses to information, motivation, confidence and categorical as Body Discrepancy (scores greater or less than 1) versus No Body Discrepancy (scores 0 or ±1) to describe the sample and test survey and message feasibility.

Dietary Restraint: Participants responded to 6 questions in the Concern for Dieting Subscale from the Dietary Restraint Scale [85]. Scores could range from 0–19. The dietary restraint score was tested for reliability using Cronbach’s Coefficient alpha (α = 0.83). The score was split at the median (8) for analyses examining differences in survey and message evaluation responses based on level of restraint to indicate young adult woman who were high or low in dietary restraint.

Knowledge Scores: Participants responded to 11 questions on knowledge of physical activity and diet. These questions were based on predetermined health misconceptions and misinformation of college students found in the literature [72], and the concepts were addressed in the in the tailored messages. For each question, the participant selected their level of agreement, scored as −2 (Strongly Disagree) to +2 (Strongly Agree). True/False questions were scored so the correct answers received a value of 1, and incorrect a value of 0. Scores were summed to create a knowledge score, with a maximum score of 15.

### 2.4. Liking Survey and Tailored Message Program

A proxy of physical activity and dietary behaviors was captured using a previously validated, online liking survey for college-aged individuals [46,82,83]. Each activity, food or beverage item was each shown as an image and text label to the left of a horizontal, hedonic scale with five faces and corresponding descriptors of “love it”, “like it”, “it’s okay”, “dislike it”, and “hate it”, and a slider allowing a continuous rating from ±100. Students were able to move the marker anywhere on the slider containing five faces: “love it”/“hate it” had a midpoint value of ±80, “like it”/“dislike it” a midpoint value of ±40, and “it’s okay” as 0. Students were able to select “never tried or done” for any of the activities, foods, and beverages.

Students were oriented to the liking survey by reporting their liking/disliking for generally pleasant and unpleasant experiences (seeing family and friends, receiving a compliment, going on vacation, taking an exam, zoom class, and being caught in a lie). Following orientation, students rated liking/disliking of physical activities (19 items), sedentary activities (5 items), and foods and beverages (47 items). The physical activities represented four categories: aerobic training, resistance training, flexibility training, behavioral inclinations. Behavioral inclinations included general habits related to physical activity preferences such as working up a sweat, exercising alone/with a partner, taking the stairs, going to the gym, attending group classes, and playing sports. Reported liking of physical activities and behavioral inclinations were averaged together to create an overall liking of physical activity score. The foods and beverages represented major food groups (vegetable, fruit, whole grains, heathy fat, low-fat dairy, refined grains, high fat protein, unhealthy fat, salty foods/snacks, sweets, and sugar-sweetened beverages), with at least three items per group.

The messages were tailored to the average liking/disliking of activity and food groups and the responses to intuitive eating, stress, and sleep questions to be motivating or reinforcing as shown in Table 1 [72]. All messages were pilot tested with a small group of college students and were edited based on their feedback [72]. The criteria for receiving a tailored messages as motivating or reinforcing were based on liking responses following our previous studies with young adults [46,82,83], our tailored message program [31,32], and the literature [86,87,88]. For example, participants who reported a high liking of a healthy item or low liking of a less healthy item received a reinforcing message encouraging the participant to continue the behavior. Participants who reported a low liking of a healthy item or high liking of a less healthy item received a motivating message. The health behavior messages (intuitive eating, stress, sleep) were tailored using participant response to validated questionnaires by criteria reported previously [86,87,88]. The motivating messages also were tailored to the participant’s preferred learning style [92] for either autonomous support or directive support. Two generic health messages were also created to serve as comparison with the tailored messages [72]. Algorithms were embedded within Qualtrics to assure each participant received 5 messages, including 4 tailored messages (reinforcing or motivating), and 1 generic message (randomly assigned from 2 possible). Two of the tailored messages were food-based messages (vegetable, fruit, whole grains, lean protein, fats, hydration, sweets, salt), one physical activity-based, and one health behavior-based (intuitive eating, stress, sleep).

### 2.5. Feasibility Measures

Participants rated the feasibility of the survey and the overall acceptability and usefulness of all the messages collectively, as well as provided responses to each message following the IMB model.

Prior to receiving their tailored messages, participants used the sliding hedonic scale to report their level of agreement/disagreement to the survey acceptability and usability questions [93]. Acceptability questions included: (1) I could answer the questions quickly and (2) I would recommend this survey to a friend. Usability questions included: “The survey was helpful in reflecting on my current behaviors”, and “The survey questions were relevant to me as a college student”. The hedonic scale was labeled with five faces with corresponding descriptors of “strongly agree”, “agree”, “neutral”, “disagree”, and “strongly disagree”, with ability to slide the marker anywhere to produce a value ±100.

Following each tailored message, participants completed four questions assessing the IMB constructs to the message and target behavior of the message: (1) interesting and specific Information learned (2 questions); (2) Motivation; and (3) Behavioral Skill (i.e., confidence) for the targeted behavior. Participants responded on the same hedonic scale with facial label (±100) specific to the displayed tailored message/behavior and text to indicate information agreement, motivation, and behavioral skills as shown in Table 2. Due to participant responses pooling around the scale labels, they were compressed to the label value, creating a 5-point scale (Table 2), and then used to create composite scores of Information, Motivation, and Behavioral Skills constructs. First, responses to the message target behaviors, including food, physical activity, other health behaviors, were averaged separately (e.g., average information for food-based messages, average motivation for physical activity message, average behavior skill for health behavior messages, etc.) and then together to create an overall information, motivation, and behavioral skill variable. For example, the average response to information for food, physical activity, and health behavior messages was averaged to create a composite information variable. Reliability of each composite variable was tested using Cronbach’s Coefficient alpha and produced sufficient reliability (<0.6–0.9’s).

Following the individual display and evaluation of messages for information, motivation, and behavioral skills, participants reported the general impressions of all 5 messages using a 5-point rating from strongly agree to strongly disagree. These questions served as on overall evaluation of participants’ agreement to learning new information about food and exercise, motivation to make a behavior change, and ability to accomplish behavior change after reading the messages. In addition, participants reported their agreement in relevancy of the messages to their experience as a college student.

### 2.6. Statistical Analysis

Data were analyzed using SPSS statistical software (Version 28, Chicago, IL, USA) with a significance criterion at *p* < 0.05. Descriptive statistics were used to analyze participant demographics and variability in IMB-based variables (knowledge scores, information, motivation, and behavioral skills measurements). Composite variables were tested for reliability using Cronbach’s alpha (diet restraint, information, motivation, behavior skills). Descriptive statistics and were used to examine responses to liking/disliking of food and physical activity items, other health behavior questions, and feasibility measures. Pearson Chi-Square statistics were used to examine differences in survey and message feasibility between participants with and without body size discrepancy, high/low levels of dietary restraint, and differences in IMB constructs between the message types. Linear regression analysis was used to test the influence of body size perception and dietary restraint on participant responses to information, motivation and behavioral skills for both message types combined, reinforcing, and motivating messages. Covariates (where appropriate) included age, race/ethnicity, and self-reported history of diagnosed eating disorder.

## 3. Results

### 3.1. Participant Characteristics

Table 3 displays the characteristics of the 189 college women who completed the online tailored health messaging program. The sample was mostly young and normal weight with an average age of 20.8 ± 0.18 and reported BMI of 23.6 ± 0.37. Most women identified as White (69.3%) and not Hispanic/Latino (83.1%). There was good representation across academic year (student status). Participant body size perception fell within the normal weight body figure range [84], with 65.1% reporting little to no body size discrepancy. Average dietary restraint was 8.1 (0–19), indicating that most of the participants had moderate level of dietary restraint.

### 3.2. Variability in Responses

The sample had good variability in liking/disliking ratings across the food and activity groups (Figure 1). Pleasant activities and being caught in a lie (i.e., unpleasant item) were included to provide context for the liking responses. Refined grains were the most liked, while high fat protein the least liked (Figure 1). Physical activity was generally rated as “It’s Okay” to “Like it”. Overall, the less healthy food items (e.g., refined grains, sweets, salty foods/snacks, unhealthy fats, sugar sweetened beverages) were liked more than the healthier food items (e.g., physical activity, vegetables, whole grains) and sedentary activities were liked more than physical activities. Internal reliability of the individual food groups and activity groups ranged from below acceptable (alpha < 0.6, *n* = 7) to acceptable (alpha ≥ 0.6, *n* = 5).

Intuitive eating responses were variable within the sample, with an average score of 22.5 ± 0.3 (range 11–34), suggesting a moderate amount of intuitive eating behaviors within this sample of young adult women. Additionally, this sample experienced moderate to high stress (91.5%) and inadequate sleep, with an average scores of 11.8 ± 0.19 (range 6–18).

Knowledge Scores: Figure 2 displays the variability in knowledge scores, showing a negative skewness impacted by 3 outliers (low knowledge scores). Examination of the quartiles revealed that many participant’s knowledge scores ranged between 10–13 (25th–75th quartile), suggesting an overall low variability in the sample.

### 3.3. Survey Evaluation (Acceptability and Usefulness)

The study sample of college women found the online survey acceptable and useful. Nearly all (92%) reported at least agree that they could answer questions quickly and found survey questions to be relevant to them as a college student. Slightly fewer women (82.6%) at least agreed the survey was helpful in reflecting on current behaviors and 80% with recommending the survey to a friend. No significant differences were found in survey acceptability and usefulness among women with/without body size discrepancy and high/low levels of dietary restraint.

### 3.4. Responses to Information, Motivation, and Behavioral Skills

Descriptive statistics are displayed for responses to information, motivation, and behavioral skills (IMB constructs) for all messages (Table 4). Each construct ranged from acceptable to very good internal reliability and good range. With both message types combined, average response to information measures (both interesting and specific) categorized as “agree” to learning interesting information and specific information from messages. Average response to motivation measures categorized as “love to” continue or try behaviors suggested in messages. Average response to measures categorized as “very confident” in continuing or trying behaviors suggested in message. Similar ratings were seen in reinforcing or motivating messages.

As shown in Table 5, there was variability in response to the messages for each IMB construct (information, motivation, behavioral skills). Frequency in responses to interesting information and specific information learned were similar between reinforcing and motivating types of messages, with 60–74% responses in agreement to learning interesting or specific information. For motivation, there was an overall significant difference between responses to reinforcing and motivational messages. Reinforcing messages had higher (87%) agreement/strongly agree (willingness) than motivational messages (66%). A slightly higher percentage (13%) of responses were neutral in motivational compared to reinforcing messages (x^2^(2, *N* = 189) = 23.51, *p* < 0.001). For behavioral skills, there was a significant difference between responses to reinforcing and motivational messages (x^2^(2, *N* = 189) = 3.91, *p* < 0.05). Very few (<4%) participants reported lack of confidence to try or continue the behavior, with 90% reporting at least confident. Higher percentage was seen in reports of high confidence to reinforcing (68%) compared to motivational messages (53%).

### 3.5. Message Evaluation

This sample of young adult women rated the overall messages as generally acceptable and relevant to them as college students, with 60% reporting “agree” or higher to learning new information and 80% “agree” or higher to message relevancy. Slightly less than half of the sample of young adult women reported agree or higher to being motivated to and confident in their abilities to accomplish the behaviors in the messages, 48.7% and 47.1%, respectively. There were no significant differences in overall collective message evaluation among participants with/without body discrepancy or with high/low level of dietary restraint.

### 3.6. Influence of Body Discrepancy and Dietary Restraint on IMB Construct Responses

Body discrepancy did not have a significant relationship with the knowledge scores acroos the sample. However, dietary restraint was a significant predictor of knowledge scores (F(1, 187) = 4.144, *p* < 0.05), where a slight increase in dietary restraint was associated with increases in knowledge scores. Higher dietary restraint scores correlated significantly but weakly with knowledge scores (Pearson r = 0.147, *p* < 0.05). Visual analysis of the relationship showed a group of women who reported a higher diet restraint and higher knowledge scores.

Neither body size discrepancy nor dietary restraint showed significant relationships with the information measurements (i.e., interesting/specific information learned) for both types of messages combined. However, dietary restraint trended on significance to predict specific information learned (F(1, 187) = 2.550, *p* = 0.07). Further examination of reinforcing and motivating messages separately also did not result in significant associations with information, yet motivating messages trended on significance with dietary restraint positively predicting motivation measures (F(1, 174) = 2.512, *p* = 0.081).

Body size discrepancy (F(1, 187) = 4.921, *p* < 0.05) and dietary restraint (F(1, 187) = 3.93, *p* < 0.05) were significant predictors of motivation responses across reinforcing and motivating messages. A slight increase in either body size perception or dietary restraint predicted an increase in motivation. However, these factors only accounted for 2.1 to 2.6% of variability in the responses. When examining reinforcing and motivating messages separately, the relationship between body size discrepancy and motivation only was seen for reinforcing messages F(1, 154) = 6.767, *p* < 0.05), and accounted for 4.2% of variability (adjusted R^2^ = 3.6%). There was no significant relationship seen between body size perception and motivating messages. In motivating messages, dietary restraint trended on significance to positively predict motivation (F(1, 174) = 3.96, *p* = 0.067). There was no significant relationship seen between dietary restraint and reinforcing messages.

In both message types combined, body size discrepancy was a significant predictor of behavioral skills responses (F(1, 187) = 4.283, *p* < 0.05), accounting for 2.2% of variability (adjusted R^2^ = 1.7). No relationship was seen between dietary restraint and behavioral skills for both message types combined. Neither body size perception nor dietary restraint significantly predicted behavioral skills responses in motivating messages. In reinforcing messages, body size discrepancy was a significant predictor for behavioral skills (F(1, 154) = 6.730, *p* < 0.01), and accounted for 6.1% of variability in responses (adjusted R^2^ = 5.4%). No significant relationship was seen between dietary restraint and behavioral skills in reinforcing messages.

Overall, these results suggest that body size discrepancy and dietary restraint have a small influence on some response measures (mainly motivation and behavioral skills). Accordingly, it can be inferred that the survey and response measures are able to capture variability in responses, partially supporting the hypothesis.

## 4. Discussion

Findings from the present study, conducted during the COVID-19 pandemic, demonstrated the feasibility of an online tailored messaging program (survey and tailored messages) aligned with behavior change theory in 189 young adult college women. The survey and tailored messages were deemed acceptable and useful, evidenced by the women reporting high agreement to learning new information, being motivated, and confident in their abilities to accomplish behaviors targeted in messages. Variability was displayed in the baseline knowledge scores of participants, information learned, motivation, and confidence responses to both motivating and reinforcing messages. Body size discrepancy and level of dietary restraint had only small effects on the participants’ level of knowledge, motivation, and confidence. Overall, this program demonstrated applicability for use in communicating tailored health recommendations for general health promotion efforts for college women, especially during a stressful period that was found to impact many health behaviors [9,10,11].

Despite our comprehensive marketing strategy for diverse recruitment, the sample predominantly identified as White, with an average age of 20 years. Height and weight were self-reported to calculate an average BMI of 23.6 kg/m^2^, which is similar to a UConn sample of young adult college women recruited prior to the pandemic [46]. Most of the women did not have a large discrepancy in their body size perception, contrary to the expected higher body size discrepancies reported in the literature [73,74]. The sample displayed a moderate level of dietary restraint, which is similar to a pre-pandemic sample but with a different dietary restraint measure [46]. Thus, the results from the present study may only be generalizable to college women who do not have high risk of excessive adiposity or disordered eating and should be considered as primary or secondary prevention efforts to promote healthier physical activity, diet quality, and other behaviors such as stress and sleep.

The acceptability and usefulness of the survey with tailored messages were equivalent to previous online tailored messaging programs in children alone in a school setting [32] and with their parents/caregivers in a clinical setting [31]. Liking of food items resembled dietary intakes observed in college adults, with higher liking of unhealthier food items suggesting higher intake of nutrient dense foods and risk of not meeting dietary guidelines [94,95,96,97]. Physical Activity was generally not liked with scores averaging between “It’s okay” and “like it”, suggesting that available activities or environment for these activities may be insufficient to be liked enough to compel physical activity behavior in college women [1,8,9,10,11]. Responses to intuitive eating, stress, and sleep resemble what is expected in college students [98,99,100,101]. 

The baseline knowledge scores in this sample of college women suggested a range in misinformation and ability of questions to capture variability in response. However, most women (75th quartile) had scores > 10, suggesting higher health behavior knowledge compared to previous literature reports [5,102,103,104]. Participants with low knowledge scores were considered outliers, and if removed would decrease the range, thus limiting the variability seen. It is possible that participants who elected to take the survey were health seeking with good health behavior knowledge, indicating that questions may have been too easy. However, evidence suggests that though participants may have knowledge and understanding of the importance of healthy behaviors; it does not always translate into behavioral skill and action [6,102,105,106,107], further supporting need for tailored health interventions.

The variability in responses to the information measures (i.e., interesting and specific) are consistent with past study findings assessing health behavior knowledge. The high agreement to learning information seen in women who received reinforcing messages suggests that, although one may be practicing a behavior, information can be improved [5,6,102,105] and motivate them to continue the behavior, as theorized in the Information Motivation Behavior Skills Theory [59]. The observed agreement to interesting and specific information learned to the motivating messages support the underlying structure of this theory. Information has an influencing relationship on motivation and behavior [59], thus can be inferred that if an individual received a motivating message due to low engagement in targeted behavior, lack of information could be a contributing factor. Although not significant, specific information had slightly higher response agreement than interesting information, suggesting acquisition of the intended information of the message [72]. Based on these results, asking if specific information was learned was the best method to measure the information construct. 

Previous literature suggests challenges to assessing the information construct of the Information Motivation Behavioral Skills model. Traditional and common measures have included knowledge questions to specific behaviors [61,64,65,69] or a single general information measure [62]. The present study employed both specific and general information measures. More novel methods of the information construct include cognitive function [58] or qualitative evaluation [66,71]. Alternate measures, such as “food literacy” may increase precision as they measure proficiency in nutrition knowledge, and employing the use of functional knowledge tests to assess behavioral skills [108]. Measuring health promotion literacy, including food literacy, has demonstrated associations with healthy eating habits [109].

The higher frequency of neutral ratings in the motivating type messages, indicative of participant willingness (i.e., motivation) to try the targeted behavior, suggests the need for intervention to move people along the stages of change [110]. Higher willingness (i.e., motivation) ratings in response to the reinforcing messages support that participants were likely practicing the healthy behaviors and were eager to continue them. Within the IMB Model, motivation influences both behavior and behavior skills [59]. Reinforcing feedback can encourage motivation and continued liking of and engagement in healthy behaviors [111,112]. Willingness to try a healthier behavior in response to a motivating message can be the focus of an intervention beyond the tailored message program, including goal setting and follow-up to support achievement of the goal.

The present study observed a higher percentage of neutral confidence ratings for motivational messages than reinforcing. The high confidence observed for reinforcing messages may be indicative that the participant has the confidence or self-efficacy to continue the healthy behavior [111,112,113,114,115]. Conversely, if an individual was not engaging in a behavior, self-efficacy or low confidence could be likely a barrier [113,114,115,116]. Other behavioral techniques may be necessary to help increase confidence [110,116] through goal setting and addressing barriers to behavior change.

Though dietary restraint and body size discrepancy had limited influence on knowledge scores, motivation, and behavioral skills in the present study, our findings are consistent with previous literature reports. For example, higher dietary restraint, or cognitive control of eating, appears to associate with a greater level of nutrition knowledge [117,118,119], consistent with the present study. Body size perception has been found to influence motivation for eating and physical activity behaviors [21,73,74,75,76,77,78,79,80,81], aligned with the significant associations seen in our sample between higher body size discrepancy and increased motivation to try or continue healthier behaviors. Our study finding of higher body size discrepancy in association with increased confidence in behavior skills adds to the mixed findings of body size effects on self-efficacy in the literature. Some studies report higher body sizes are associated with less engagement in healthy behaviors [120,121] due to low self-efficacy related to body size, experienced weight stigma/bias, discouragement, or fear of failure [19,79,80,81]. Consequently, high discrepancies can lead to maladaptive behaviors, or compensatory behaviors in young women where over exercise or undereating becomes common [17,18,19,20,21,78]. However, in our sample higher body size discrepancy was significantly associated with increased confidence only in responses to reinforcing messages, further supporting the feedback relationships of motivation, skill, and successful engagement discussed earlier [111,112,113,114,115]. Nevertheless, the significant relationships observed between dietary restraint and body discrepancy with the IMB constructs only accounted for a small percentage of variability in responses. These findings support the feasibility of our survey with tailored messages program for college women who report low risk of excessive adiposity or disordered eating to encourage health promoting physical activity and diet quality.

The study does present several limitations. Due to this being a feasibility study of a smaller sample size, it was not powered to make inferences from the statistical analyses. Although, we implemented a comprehensive marketing plan, recruitment methods were solely virtual due to the University COVID-19 precautions and may have limited our ability to obtain a diverse sample as evidenced by the limited racial/ethnic and body size diversity seen. In person methods may assist in developing trustworthy relationships that can enhance communication and recruitment of less represented populations [122,123,124]. Although sample characteristics were reflective of many University demographics [16], lack of adequate representations of racial/ethnic minority populations cautions generalizability of findings. Results should only be applied for consideration in health promotion programs targeted for a low-risk groups. Future methods in stakeholder development/ communication, recruitment, tailoring of information, and inclusion of multilevel interventions may be necessary to improve program delivery [123,124,125]. The survey relied on self-reported data, which always presents risk of bias. Nonetheless, utilizing liking as a proxy of behavior has been demonstrated to limit bias in response [43,44,45,46]. Another limitation was the degree of randomization for the message delivery. While messages were initially randomized, the algorithms were set for each participant to receive one general message, one physical activity message, 2 food-based messages and one other health behavior message. This is a potential limitation as a participant could have had a higher need to address another behavior over the behavioral message they received. For example, the participant may have had a higher need to address multiple food behaviors over a physical activity behavior. Lastly, although responses to information, motivation, and behavioral skills were combined for each message to equate to 4 responses for each measure for 1 participant, only 1 question was used to measure the constructs. This could limit comparability to other studies that use various validated scales to measure the constructs.

Despite the limitations, there are several strengths to the study. Only using 1 question to measure each construct can strengthen results as the questions were specific to the intended information/behavior versus generalized in many previous studies. Secondly, using liking to measure and trigger the tailored behavior strengthened methods and study results. Differences seen in responses to motivational and behavioral skill measures in the two types of tailored messages (reinforcing, motivational) support the use in liking as a proxy of behavior. Another strength is the ease and accessibility of the online delivery of the survey and messages. The online delivery allowed participants to complete the survey on their own time and at their own pace with relatively low time commitment. This was especially important during the online nature of university classes and programs during COVID-19 and assisted greatly with the distribution of the program during this time. Online delivery also allows for further reach to students in different academic programs. The online program allowed for immediate delivery of information to the participants and researchers. In addition, the online nature allowed for rapid adaptation of the survey and tailored health messages. Although the length of the online program averaged 25 min, it provided participants with information consistent with a nutrition professional as tailored recommendations were provided. This length is shorter than the typical 1 h duration of initial appointments and 30 min follow up appointments with nutrition and physical activity professionals. This demonstrates the future applicability of this program in a counseling setting. The program can be adapted for use as a pre-appointment tool, in between appointments for support, or even in place of appointments for patients who may only need general healthy eating and behavior recommendations. Additionally, the program can be adapted for used as a wide scale campus effort to survey and improve the general health behaviors of student populations. The focus of tailoring in this program can help to increase relevancy to health information, decreasing previous barriers found to traditional health promotion efforts in young adults [25].

## 5. Conclusions

The results supported the feasibility of the online survey and tailored message program to promote healthier diet and physical activity for college women. The program aligned with a theoretical framework focused on the information, motivation, and confidence needed to follow healthier behaviors. College women found the survey and messages acceptable and useful. There was variability in response to each message for information learned as well as motivation and confidence to follow healthier behaviors, with minimal effects of the participant’s body size perception and level of dietary restraint on these responses. The information gained from the responses to the survey and tailored messages can provide direction for further individualized interventions as well as broader campus efforts to promote healthier diets and physical activity.

## Figures and Tables

**Table 1:** Tailored Message Categories and Examples.

Category	Composite Group	Items	Message Category	Message Example
Physical Activities	Aerobic Training	Walking, running, sprinting, high intensity interval training, playing sports, biking, circuit training	Physical Activity	
	Resistance Training	Barbell exercises (squat, deadlift, bench press), free weights, cable exercises		Keep up with the great movement you’re doing! Setting timers to do quick stretches or air squats can help to increase physical activity levels. (Reinforcing)
	Flexibility Training	Pilates, yoga, flexibility training		
	Behavioral Inclinations	Exercising alone, exercising with others, going to the gym, taking the stairs, instructor-based classes, working up a sweat		
Sedentary Activities	Sedentary	Watching TV/Streamed channels, scrolling through phone/social media, playing video games, using computer, reading	Physical Activity	Try creating a habit of setting a timer to get up and move. Small movements like squats or doing a fun activity help to increase physical activity. (Autonomous Motivating)
**Foods**	Vegetables	Broccoli, carrots, greens, tomatoes, sweet potato, mushroom	Vegetables	Vegetables are a great source of fiber. Try using the salad bar to add vegetables to meals to eat at least 2 cups a day. (Autonomous Motivating)
	Fruit	Melon, strawberries, blueberries, pineapple	Fruit	Choose Fruit! Fruits are packed with vitamins and minerals that make your skin glow. Eat at least 2 cups or piece of fruit a day. (Directive Motivating)
	Whole Grains	Whole wheat bread, oatmeal, granola, shredded wheat cereal	Whole Grains	Great job! Whole grains are a great source of dietary fiber and B vitamins, which support a healthy digestive system and energy metabolism. Try a whole grain bowl with quinoa or brown rice and your favorite add ins. (Reinforcing)
	Healthy Fat	Tuna, baked white fish, olive oil	Heart Healthy Fat	Great job on choosing heart healthy fats. Foods like nuts, avocado, salmon, & olive oil nourish your body. (Reinforcing)
	Refined Grains	White rice, bagels/rolls, spaghetti/pasta, snack crackers, pizza	Whole Grains	Whole grains are a great source of dietary fiber and B vitamins, which support a healthy digestive system and energy metabolism. Make a whole grain bowl with quinoa or brown rice and your favorite add ins. (Directive Motivating)
	High Fat Protein Foods	Hot dog, fried chicken, bacon, fast food	Lean Protein	Try to select a variety of lean protein foods to improve nutrient intake. Sources like chicken, fish, eggs, and beans, help to build a strong body. (Autonomous Motivating)
	Unhealthy Fat	Cheddar cheese, mayonnaise, full fat dressing, whole milk	Heart Healthy Fat	Healthy fats are good for your heart. Select foods like nuts, avocado, salmon, & olive oil to nourish your body. (Directive Motivating)
	Salty Foods/Snacks	Salty snacks, noodle soups, French fries	Salt	Reading a nutrition label is a great way to reduce salt intake. Continue limiting salt by choosing foods ≤ 140 mg of sodium.
	Sweets	Ice cream, cookies/cake/pastries, cake icing/frosting, cheesecake	Sweets	Feel like you have a sweet tooth? When enjoying sweets, try to make each bite satisfying by taking your time and enjoying every bite! (Autonomous Motivating)
	Sugar Sweetened Beverages	Chocolate milk, soda, flavored coffee drinks	Hydration (Water)	Sugary beverages can lead to dehydration which can cloud our thinking and make us tired. Drink a glass of water every hour to stay hydrated. (Directive Motivating)
**Health Behaviors**	Intuitive Eating	7 Questions from Intuitive Eating Scale (Scored from Strongly Disagree to Agree) [86]	Intuitive Eating	Your body knows best! Continue to eat intuitively by listening to your body’s hunger and fullness cues to stay within the green areas for most meals and snacks. (Reinforcing)
	Stress	Within the last 30 days, how would you rate the overall level of stress you have experienced? [87]	Stress	In times of high stress, try to take a few deep breaths. Deep breathing has proven to be effective in calming oneself. (Autonomous Motivating)
	Sleep	4 questions from the Pediatric Daytime Sleepiness Scale (adapted to College Students) [88]	Sleep	Sleep is important for your mental and physical health. Before bed, stretch, reflect, and shut off all screens to improve your sleep. (Directive Motivating)

**Table 2:** Information, Motivation, behavioral skills (confidence) message response recodes.

Information Labels	Motivation Labels	Behavioral Skills Labels	Original Ranges	Compressed Scale	Interval Range (for Means)
I learned a new or interesting fact from this message. I learned [insert targeted specific fact]	How much would you like to engage in/continue [targeted behavior]?	How confident are you that you can engage/continue [targeted behavior]?			
strongly disagree	hate to	Not all confident	−61 to −100	1	1–1.80
disagree	dislike to	Somewhat confident	−21 to −60	2	1.81–2.60
neutral	neutral	Moderately confident	−21 to 20	3	2.61–3.40
agree	like to	very confident	21 to 60	4	3.41 to 4.20
Strongly agree	Love to	completely confident	61 to 100	5	4.20 to 5.0

**Table 3:** Characteristics of 189 Young Adult Women.

Category		%
**Age**	17–20	52.4
	21–24	40.2
	25+	7.4
BMI Categories *	Underweight	6.3
	Normal Weight	62.4
	Overweight	17.5
	Obese Class I	4.8
	Obese Class II	3.7
	Obese Class III	0.5
**Race**	Asian	15.3
	Black/African American	6.3
	White	69.3
	Other	9
**Ethnicity**	Hispanic/Latino	16.9
	Not Hispanic/Latino	83.1
**Student Status**	First-year student	19.0
	Sophomore	17.5
	Junior	21.2
	Senior	27.5
	Graduate Student	13.2
	Other	1.6
**Body Size Perception ^+^**	No Body Size Discrepancy Body Size Discrepancy	65.1 34.9

* Calculated using self-reported height and weight. BMI Categories are as follows: Underweight ≤ 18.5; Normal Weight = 18.5–24.9; Overweight = 25.0–29.9; Obese Class I = 30.0–34.9; Obese Class II = 35.0–39.9; Obese Class III = >40; **^+^** Participants selected which labeled figure matched their current and ideal body size from 1 (smallest) to 9 (largest); Body Size Perception (BSP) was defined by current-ideal body image. Body Size Discrepancy present if BSP > 1 or <−1.

**Table 4:** Descriptive Statistics of IMB Construct Responses for All Messages ^†^.

Construct	Min	Max	Mean	St Dev	St Error	Cronbach’s Alpha
Interesting Information	1	5	3.46	0.98	0.071	0.82
Specific Information	1	5	3.87	0.89	0.064	0.87
Motivation	2	5	4.47	0.57	0.041	0.71
Behavioral Skills	1.33	5	3.99	0.82	0.06	0.66

^†^ Mean values between 2.61–3.40 = “neutral”, “moderately confident”; 3.41–4.20 = “agree”, “like to”, “very confident”; 4.20–5.0 = “strongly agree”, “love to”, “completely confident”.

**Table 5:** Number of participants (*n* = 189) who fell into each response category for message types that were reinforcing or motivation according to information, motivation, and behavioral skills.

Interesting Information	Strongly Disagree	Disagree	Neutral	Agree	Strongly Agree
Reinforcing	14	30	29	76	40
Motivational	15	30	34	80	30
**Specific Information**					
Reinforcing	11	13	21	87	57
Motivational	6	17	27	89	50
**Motivation ^†^**	**Hate to**	**Dislike to**	**Neutral**	**Like to**	**Love to**
Reinforcing	3	6	15	61	104
Motivational	9	17	39	64	60
**Behavioral Skills ^†^**	**Not at all** **confident**	**Somewhat** **confident**	**Moderately** **confident**	**Very** **confident**	**Completely** **confident**
Reinforcing	1	5	5	49	129
Motivational	0	6	17	66	100

**^†^** Sum of the highlighted categories significantly different than unhighlighted categories with in a message type by chi square testing.

## Data Availability

The data presented in this study are available on request from the corresponding author.
